# Genome Sequences of Two Nondomesticated *Bacillus subtilis* Strains Able To Form Thick Biofilms on Submerged Surfaces

**DOI:** 10.1128/genomeA.00946-14

**Published:** 2014-09-25

**Authors:** Pilar Sanchez-Vizuete, Kosei Tanaka, Arnaud Bridier, Yusuke Shirae, Ken-ichi Yoshida, Théodore Bouchez, Stéphane Aymerich, Romain Briandet, Dominique Le Coq

**Affiliations:** a INRA, UMR1319 Micalis, Jouy-en-Josas, France; b AgroParisTech, UMR Micalis, Jouy-en-Josas, France; c Organization of Advanced Science and Technology, Kobe University, Kobe, Japan; d IRSTEA, UR HBAN, Antony, France; e Department of Agrobioscience, Graduate School of Agricultural Science, Kobe University, Kobe, Japan; f CNRS, Jouy-en-Josas, France

## Abstract

Genomes of two nondomesticated strains of *Bacillus subtilis* subspecies *subtilis*, NDmed and NDfood, have been sequenced. Both strains form very thick and spatially complex biofilms on submerged surfaces. Moreover, biofilms of the NDmed isolate were shown to be highly resistant to antimicrobials action.

## GENOME ANNOUNCEMENT

*Bacillus subtilis* is a non-pathogenic Gram-positive bacterium largely used in biotechnological processes and academic research. Although this bacterium is mainly found in the soil, several strains have recently been isolated from other environments (1–6). *B. subtilis* is able to form structured biofilms in which cells are embedded in a self-produced matrix of polymers. For studying biofilm properties and the associated genetic regulation, most researchers have used the “less domesticated” strain NCIB3610 instead of the reference strain 168, which is defective for determinants involved in multicellular behavior (7). Here we present the genome sequences of two *B. subtilis* natural isolates. The first strain, NDmed, was isolated from an endoscope washer disinfector in a hospital in England (8). The second strain, NDfood, was isolated from a dairy product in France (9). Both strains are able to form spatially organized biofilms with protruding structures on submerged surfaces as well as wrinkled macrocolonies and robust pellicles (9). In addition, submerged biofilms of NDmed exhibited high resistance to antimicrobials and the ability to protect pathogens (10).

Genome sequencing of *B. subtilis* NDmed and NDfood strains was performed using Illumina Miseq technology. A total of 4,595,294 and 3,622,152 reads were obtained for NDmed and NDfood, respectively. The raw sequences generated were mapped to the reference strain 168 genome (11) and using the CLC NGS assembler, the reads were assembled into 10 contigs for NDmed (ranging from 4.9 kb to 1.2 Mb) and 12 contigs for NDfood (ranging from 1.4 kb to 1.1 Mb), with an average coverage of 271-fold and 214-fold, respectively. For both strains the total size of the assembly was around 4.06 Mb and the G+C content about 43.7%.

Comparison of NDmed and NDfood sequences to the sequence of the reference strain 168 established that these two nondomesticated isolates are very close to the reference strain 168, with less than 100 single-nucleotide polymorphisms (SNPs) and less than 50 insertions/deletions, and among which the great majority were common to both strains.

As in other *B. subtilis* natural isolates (e.g., BsP1 [1], RO-NN-1 [4], BsN5 [12]), the SPβ prophage (134.4 kb) and the conjugative element ICEBs1 (20.5 kb) are missing in NDmed and NDfood strains. However, a putative prophage (44.2 kb) is present in both strains immediately downstream of the *glnA* gene. A highly similar prophage can be found at the same position in strains AUSI98 (4) and PS216 (5). No plasmids were observed in NDmed and NDfood strains, such as the one present in NCIB3610 which encodes the ComI transformation inhibitor (13). Interestingly, the two nondomesticated strains presented here are naturally competent, which facilitates their use in research.

The genomes of NDmed and NDfood isolates could provide a better understanding of *B. subtilis* social behavior in natural environments. Although all the biofilm genes found defective in strain 168 have an identical sequence in NDmed, NDfood, and NCIB3610, other differences found between this “less domesticated” strain and the two nondomesticated isolates would shed light to their specific phenotypes.

### Nucleotide sequence accession numbers.

These two whole-genome shotgun projects have been deposited at DDBJ/EMBL/GenBank under the accession numbers JPVW00000000 for *B. subtilis* NDmed and JPVX00000000 for *B. subtilis* NDfood. The versions described in this paper are the ﬁrst versions, JPVW01000000 and JPVX01000000.
